# Comparative evaluation of the effectiveness of a novel composite bone substitute synthesized from eggshell-derived hydroxyapatite and fish collagen in bone regeneration of critical-sized calvarial defects in Wistar rats

**DOI:** 10.3389/fdmed.2025.1731880

**Published:** 2026-01-12

**Authors:** Sruthy Prathap, K. S. Rajesh, Nebu George Thomas, P. K. Binsi, Suprith Surya, M. S. Prathap, Ranajit Das

**Affiliations:** 1 Department of Periodontology, Yenepoya Dental College, Yenepoya University, Mangalore, India; 2 Department of Periodontology, Pushpagiri College of Dental Sciences, Pushpagiri Institute of Medical Sciences and Research Centre, Thiruvalla, India; 3 Fish Processing Division, Central Institute of Fisheries Technology, Cochin, India; 4 Advanced Surgical Skill Enhancement Division, Yenepoya University, Mangalore, India; 5 Department of Conservative Dentistry and Endodontics, Yenepoya Dental College, Yenepoya University, Mangalore, India; 6 Yenepoya Research Centre, Yenepoya University, Mangalore, India

**Keywords:** bone regeneration, CBCT, composite, critical bone defect, eggshells, fish collagen, hydroxyapatite, Wistar rats

## Abstract

**Background:**

Large bone deformities pose significant challenges in regenerative periodontal surgeries. A healthy underlying bone and the osteoinductive effect of the surrounding environment are the primary factors that influence bone regeneration. Collagen and hydroxyapatite are universally used bone substitutes owing to their excellent biocompatibility and biodegradability. Innovations in the osteoinductive properties of hydroxyapatite and its use in different modified forms, combining it with other materials, may result in enhanced biomechanical properties. This research utilized domestic chicken eggshells to synthesize hydroxyapatite and fish scales to extract collagen. The novel composite graft was prepared by integrating both materials. The beneficial effects of the composite graft on the healing and regeneration of bone were assessed in the calvarial bone of Wistar rats both radiographically and histomorphometrically.

**Methods:**

Hydroxyapatite was synthesized from domestic chicken eggshells using the chemical precipitation method. Collagen was extracted from the scales of Rohu fish. The novel composite material was prepared by direct mixing and lyophilization, with further mixing with glycerol to attain an appropriate consistency. After preliminary characterization, bone regeneration was assessed in surgically created critical bone defects in rats. Radiographic bone fill was assessed using cone beam computed tomography, which was followed by histomorphometry. The bone regenerative capacities of the two novel materials were compared with empty control sites and a commercially available standard bone graft (Bio-Oss).

**Results:**

The novel materials displayed significantly superior regenerative capacity both radiographically and histomorphometrically when compared to empty controls, but the regenerative ability was lower when compared to the standard bone graft material (Bio-Oss) over 90 days. A trend of increased new bone formation and significantly higher radiodensity was observed in the sites treated with the composite graft compared to the empty control (*p* < 0.0001) and eggshell-derived hydroxyapatite groups (*p* < 0.01). Moreover, Bio-Oss exhibited a marginal improvement in radiodensity compared to the composite graft (*p* < 0.05).

**Conclusion:**

The composite graft demonstrated improved bone regeneration and radiodensity in the surgically created critical-sized defects. Hence, it can be considered a promising material for the regeneration of periodontal and peri-implant defects. Further laboratory and clinical studies may be required to confirm its osteoinductive properties.

## 1 Introduction

Bone has a remarkable ability for self-repair and regeneration. Yet, natural healing mechanisms may be insufficient for defects arising from trauma, congenital causes, tumors, and periodontal and peri-implant diseases. Reconstruction of bone deficiencies is still a clinical challenge. External surgical intervention and the use of regenerative materials are inevitable, especially in conditions where the capacity of the bone to regenerate is critically affected (1).

Researchers in the field of dentistry have been thoroughly investigating and developing biocompatible materials readily extracted from animals and their by-products to be used as viable bone graft substitutes. The desire to incorporate the favorable properties of different materials into a single graft compound has led to the production of various composite grafts. By incorporating different biomaterials, properties such as porosity, structural stability, osteoinductivity, and osteogenicity can be significantly improved (2).

Hydroxyapatite (HPA), the main component of human bone and collagen (COLL) the major structural protein of bone and connective tissues, is often used for the synthesis of bone substitutes. However, though it is biocompatible and osteoconductive, it is brittle, with a relatively slow rate of resorption, and lacks compressive and tensile strength (3). Moreover, the effectiveness of collagen as a standalone material may be hindered due to its inherent lack of mechanical strength and high degradation rate (4).

Thus, it is necessary to combine these materials to broaden their applications and scope. Composites prepared from hydroxyapatite and collagen have the capacity to promote bone regeneration, mimicking the natural structure and properties of bone, and these materials may have complementary effects (5).

Hydroxyapatite occurs naturally (corals, shells, bovine, or porcine sources) or can be artificially synthesized (3).

It has been suggested that eggshell exhibits favorable characteristics as a biomaterial for bone grafting, owing to its biocompatibility and osteoconductive attributes (6).

Due to the wide range of applications, collagen of mammalian origin (bovine and porcine) has been extensively explored, but religious constraints and transmission of diseases have limited its use (7). Fish collagen, due to its ease of extraction, superior level of Type I collagen content, excellent absorption properties, and biocompatibility, has attracted the attention of the research community (8).

It is also associated with a minimum risk of disease transmission from animals to humans, negligible environmental contamination, and fewer ethical and religious concerns, making it an ideal resource for product development (7). Although previous studies support the use of eggshell-derived hydroxyapatite (EHPA) and fish collagen independently for bone regeneration, there are very few studies on the combination of these two materials (4–8). Glycerin was used as a binder in one study to improve the handling of the composite graft (9).

Animal experimentation is a critical component in translational sciences and medical technology development, despite the ethical concerns and efforts to develop alternatives (10).

The rat calvarial defect method is a rapid, high-throughput method for an *in vivo* evaluation of bone regeneration (11). This study aims to evaluate the efficacy of EHPA and a composite bone substitute derived from EHPA and fish collagen (EHPA/COLL) in bone regeneration of critical-sized defects in rat calvarial bones and compare them with empty control sites and sites treated with a commercially available standard bone graft (Bio-Oss: Geistlich Pharma AG, Wolhusen, Swtzerland).

## 2 Materials and methods

Hydroxyapatite was synthesized from domestic chicken eggshells using the chemical precipitation method (12). After thoroughly cleaning and removing the membranes, the shells were dried and powdered in a domestic blender and passed through a sieve. Eggshell powder (1 g) was calcined at a temperature of 900 °C in a muffle furnace for 2 h, giving rise to calcium oxide, which was further converted to calcium hydroxide, due to its hygroscopic nature. The calcium hydroxide was weighed and mixed with distilled water to form a 0.3M suspension, and reacted with a 0.5M diammonium hydrogen phosphate solution at a stoichiometric ratio of Ca/P = 1.67 to synthesize hydroxyapatite.

Collagen was extracted from the scales of Rohu fish (*Labeo rohita*) using a laboratory-standardized protocol. The scales were subjected to a series of washing protocols, initially with 10% NaCl in the ratio of 1:10 (scale: media) for 24 h to remove any adhering debris and slime. Demineralization was carried out using 0.4M HCl in the ratio of 1:10 (scale: media) for 3 h. It was further treated with 0.5M acetic acid at pH 2.5 in the ratio of 1:8 (scale: media) for 48 h. The insoluble portions were removed by filtration, and the supernatant was collected. The residue was extracted with 1% pepsin in 0.5M acetic acid for 48 h in a ratio of 1:8 (scale: media). Both the acid-soluble and pepsin-soluble fractions were pooled. The soluble collagen was collected by salting out of the pooled filtrate using NaCl to a final concentration of 0.9M and kept undisturbed for 10 h. Collagen was collected as a pellet by centrifugation at 8,000 rpm for 1 h. The pellet was then re-dissolved in 0.5M acetic acid. This salting-out-salting-in process was repeated twice more. The salt was removed from the final suspension by dialysis (molecular weight cut-off range of 14 kDa, dialysis tubing cellulose membrane, Sigma Co, St. Louis, MO, USA) initially against 0.5M acetic acid for 48 h under stirring at 150 rpm, and subsequently with distilled water until the suspension reached a neutral pH. Purified collagen was collected by centrifugation at 4 °C for 1 h and freeze-dried. All the extraction processes were carried out at 4 °C.

The biocomposite was developed by mixing 60% HPA and 40% collagen to mimic the natural bone (13). Thus, 6 g of eggshell-derived HPA was mixed with 4 g of fish collagen powder. The composite was homogenized using a mortar and pestle for 60 min, followed by lyophilization. A Benchtop lyophilizer was used (Labconco, Kansas City, MO, USA) at a temperature of 80 °C, vacuum level of 0.02 mbar, sample volume of 5 mL, and flask size of 15 mL. The procedure was carried out for 24 h.

A binder (glycerol) was added to the product and 10 mg of the composite material was mixed with 6 mL of glycerol before application to provide an ideal consistency. The materials underwent testing using Fourier transform infrared spectroscopy (FTIR), X-ray diffraction (XRD), and Scanning electron microscopy (SEM).

Preliminary tests for *in vitro* cell viability of the materials were done using the Methyl thiazolyl tetrazolium (MTT) assay on L929 cells (14). At concentrations of 12.5, 50, and 100 µg/mL, the cell viability of EHPA was found to be 91.0141 ± 3.904, 116 ± 3.093, and 122.825 ± 2.868, respectively, whereas the composite graft demonstrated a cell viability of 79.76 ± 1.938, 121.01 ± 2.998, and 106.82 ± 2.597, respectively, at the same concentrations. As the results were found to be favorable, we planned to conduct the *in vivo* experiment.

The sample size was calculated using G*Power (version 3.1). Because no previous studies using this specific composite material were available, an *a priori* medium effect size was assumed. For a one-way ANOVA with four independent groups, the appropriate effect size metric is *f*. A medium effect size corresponds to *f* = 0.25, which is commonly recommended in biomedical and preclinical animal studies (15, 16). Using *α* = 0.05 and power =0.80, the required sample size was calculated to be six defect sites per group (total = 24 sites).

### 2.1 Intrabony defect creation in Wistar rats

#### 2.1.1 Surgical Procedure

**Figure 1:**
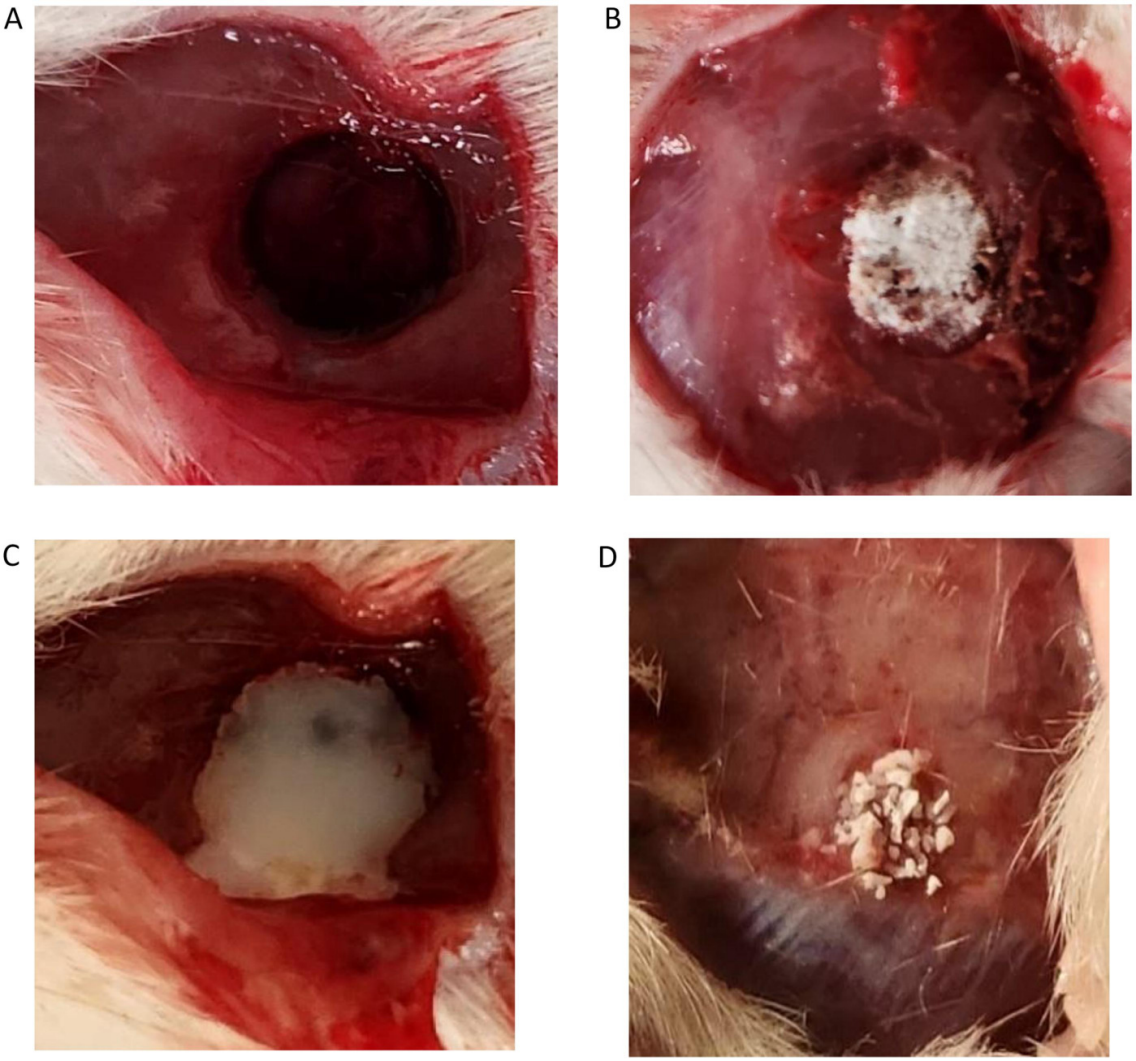
Preparation of critical-size defects and placement of different graft materials: (**A**) empty control site, (**B**) sites treated with EHPA, (**C**) site treated with the composite graft (EHPA/COLL), and (**D**) sites treated with Bio-Oss.

Ethical clearance was obtained from Yenepoya University Institutional Animal Ethics Committee (YU/IAEC/19/2023) and 24 Wistar rats were included in the study. Preoperatively, standard rat health tests were conducted in a 10-day preparation period. General anesthesia was induced with an intramuscular injection (0.5 mL/kg) of a combination of ketamine hydrochloride and xylazine. A 2-cm deep midline skin incision was made in the scalp, and the periosteum and the musculature were reflected laterally using a periosteal elevator. A 5-mm wide, critical-sized defect was created in each animal in the inner cortical half of the calvarial bone using a physiodispenser and trephine with a diameter of 5 mm at 2,000 rpm under saline irrigation. The 5 mm defect size was chosen as it does not heal spontaneously during the lifespan of the animal without leaving a hollow. There were four groups, with the first group serving as the empty control, the second group treated with EHPA, the third group treated with the composite graft (EHPA/COLL), and the fourth group treated with standard commercially available graft material (Bio-Oss: Geistlich Pharma AG, Wolhusen, Swtzerland) (Figure 1).

The wound was sprayed with an antibiotic powder, and the incision was closed with interrupted sutures (Vicryl 4-0). After the operation, all the animals received intramuscular injections (0.5 8 mL/kg) of Penicillin G. All the animals were euthanized with an intraperitoneal overdose (100 mg/kg) of pentobarbital (100 mg/mL) 90 days postoperatively. Their skulls were dissected out, and histological investigations were performed.

### 2.2 Assessment of regeneration

#### 2.2.1 Radiographic investigations

The skull, including the defect regions, was subjected to radiographic analysis using cone beam computed tomography (CBCT) after 90 days of implantation. The harvested samples were placed 7 cm above a template. The exposure settings were 90 kV, 6.3 mA, high-resolution field of view, voxel size of 0.127 mm, and exposure time of 11 s. The measurement of mean gray values was assessed with ImageJ software. The region of interest corresponding to the defect area was selected using the circle tool, as freehand selection of the area may be a potential source of measurement error. In the same image, another region of healthy bone surrounding the same area was selected. Care was taken such that the area of the healthy bone neither exceeded nor fell below the size of the defect. The selected areas were analyzed five times each by three different operators, and the mean values were calculated (17, 18).

#### 2.2.2 Histomorphometric analysis

##### 2.2.2.1 Histological processing

The calvarial bones were removed to conduct *en bloc* biopsies from the operated areas. A few specimens, along with surrounding soft tissues, were fixed with 10% neutral buffer formalin, dehydrated in increasing ethanol concentrations (70%–100%), cleared using an acetone alcohol mixture, and embedded in methyl methacrylate (MMA). After polymerization in MMA, thick coronal sections (70–100 µm) of the skull calvarial region were cut from the Poly methyl methacrylate (PMMA) block using a linear precision saw microtome (ACCUTOME 100, Struers, Copenhagen, Denmark). These sections were adhered to a glass slide, ground, and the surface was polished using a variable speed grinder polisher (ECOMET 3000, Buehler, Leinfelden-Echterdingen, Germany). These sections were stained with hot Stevenel's Blue. The stained sections were evaluated in a trinocular transmitted light microscope (Nikon DS Ri 1, Nikon Corporation, Shinagawa-ku, Tokyo, Japan).

The remaining samples were decalcified with 10% formic acid for 3 weeks, followed by washing under tap water overnight. After dehydration, the samples were embedded in paraffin (Paraffin embedding center, HistoCore Arcadia C&H, Leica, Germany) to 3–5 μm (HistoCore MULTICUT, Leica, Wetzlar, Germany) and stained using hematoxylin and eosin (H&E). A histological analysis of the newly formed bone was performed. The tissue slides were examined by a veterinary pathologist under a light microscope (Leica DM—500). The H&E-stained images were captured using Leica LAS software. The formation of new bone and fibrous tissue and other potential parameters, such as the presence of inflammatory cells and residual graft materials, were assessed. The data obtained from the computer image analysis are presented as mean values and standard deviations (SD).

## 3 Results

**Figure 2:**
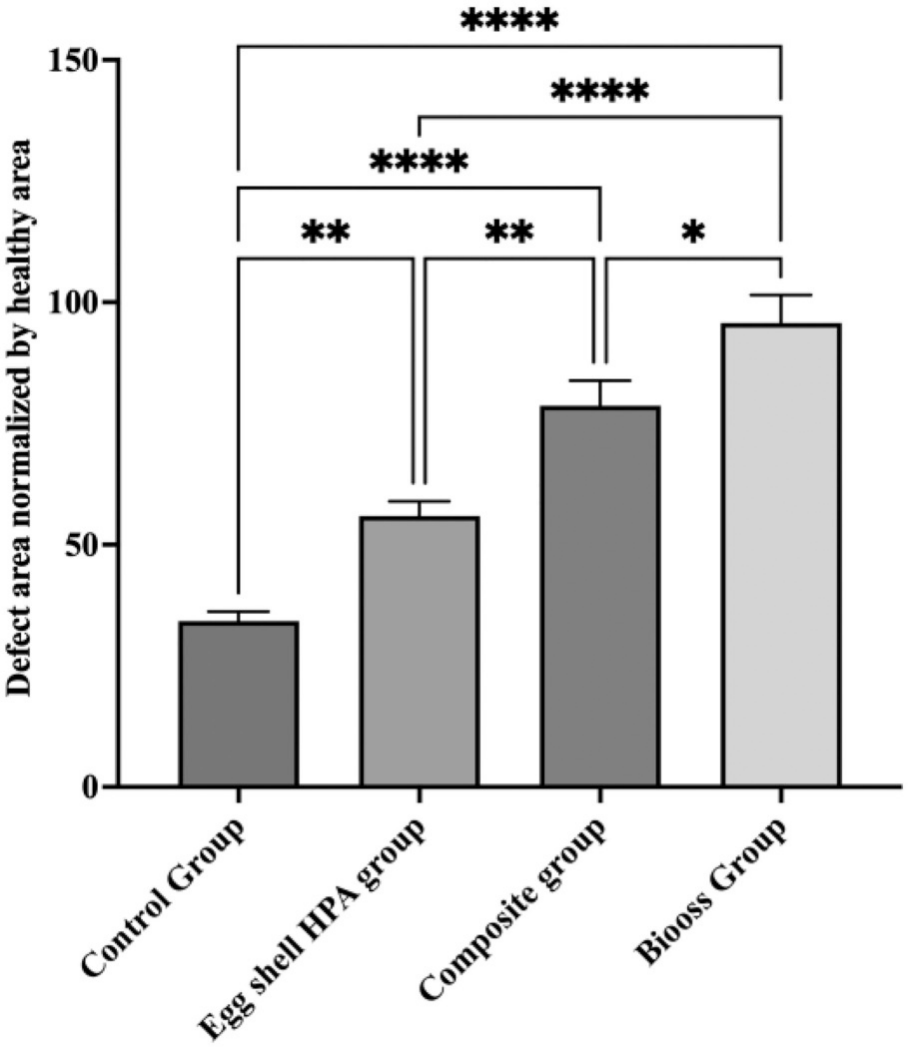
Bar graph comparing the bone densities between the groups in the ImageJ analysis.

The statistical analysis was performed using the Statistical Package for the Social Sciences version 18.0 (SPSS, Inc., Chicago, IL, USA). A value of *p* ≤ 0.05 was considered statistically significant. A two-way ANOVA test was used to compare the radiographic bone densities between the four groups. The statistical analysis demonstrated significant differences in bone density across the four groups (*p* < 0.05). Tukey's pairwise comparisons showed that the composite graft group exhibited significantly higher bone density than the empty control (*p* < 0.0001) and EHPA groups (*p* < 0.01). The Bio-Oss group showed the highest radiodensity values, which were significantly greater than the empty control (*p* < 0.0001) and EHPA groups (*p* < 0.0001). The difference between the Bio-Oss and composite graft groups was marginal but was also statistically significant (*p* < 0.05) (Figure 2).

### 3.1 Assessment of bone regeneration

#### 3.1.1 Radiographic analysis using CBCT

**Figure 3:**
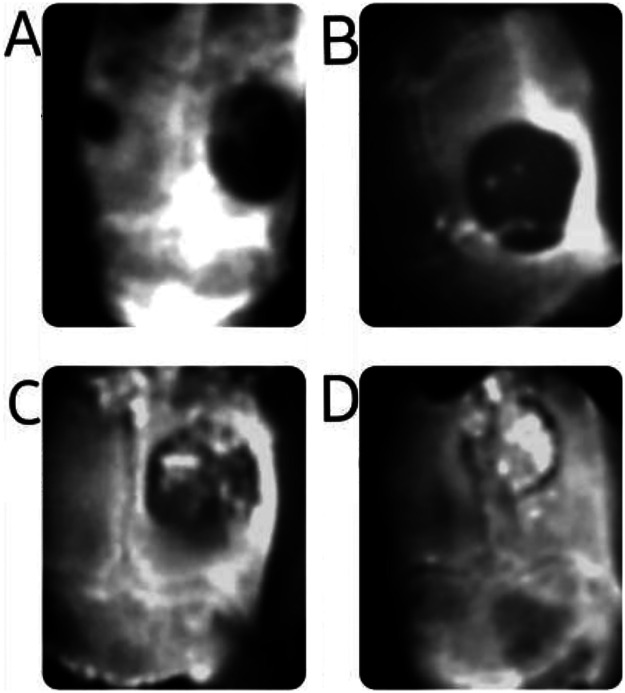
CBCT images of defects (coronal view): (**A**) empty control site, (**B**) site treated with EHPA, (**C**) site treated with the composite graft, and (**D**) sites treated with Bio-Oss.

**Figure 4:**
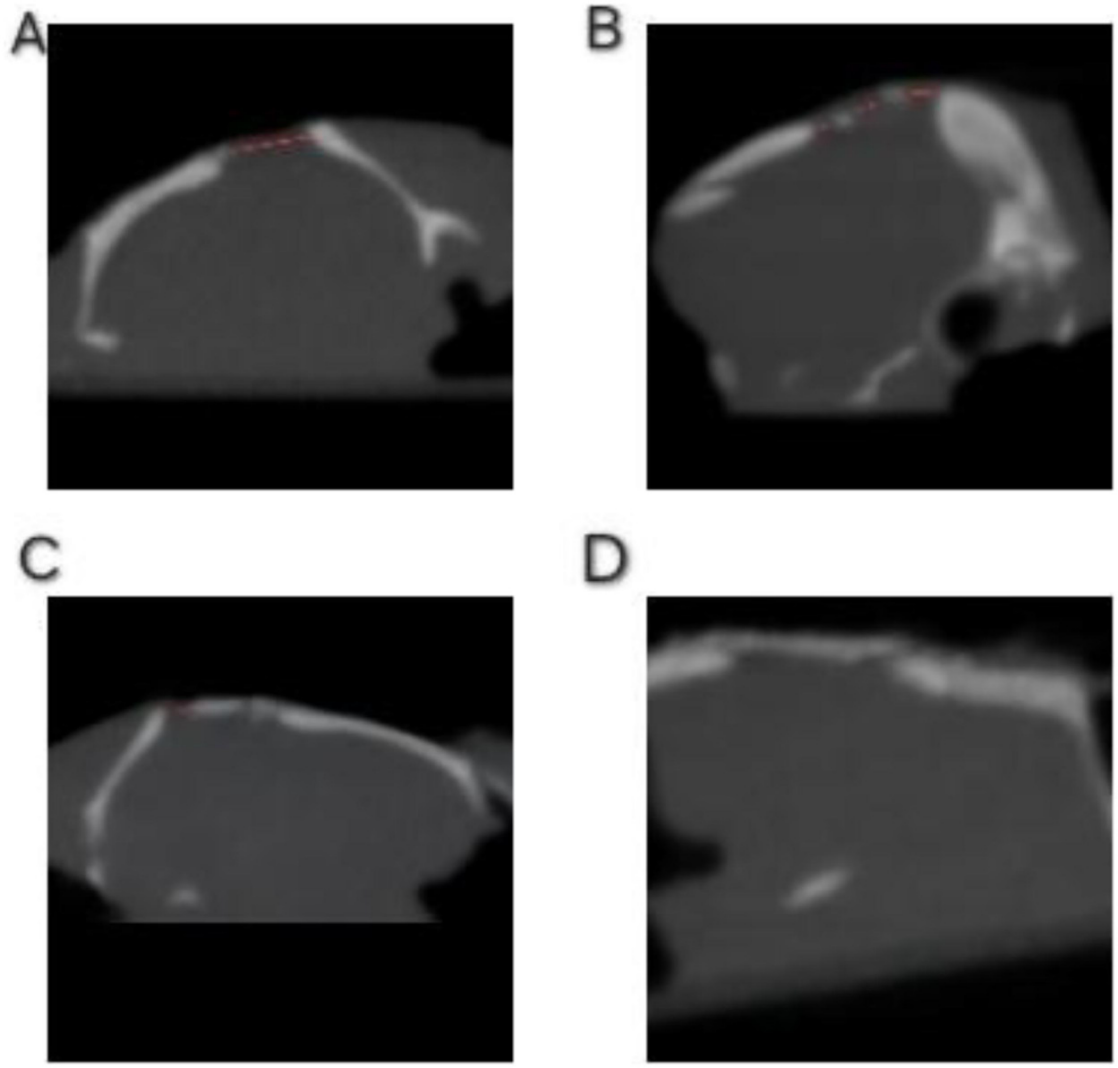
CBCT images of the defects (axial view): (**A**) empty control site, (**B**) site treated with EHPA, (**C**) site treated with the composite graft, and (**D**) sites treated with Bio-Oss. The red dots represent the radiolucent areas devoid of bone formation.

The analysis of CBCT images revealed clear differences among the groups in terms of structural changes in the graft material and progression of new bone formation (Figures 3, 4). On comparison of the coronal sections of the defects between the groups, high-density areas reaching the center of the defect were observed in all the samples in the Bio-Oss group (Figure 3). The composite group also exhibited significantly increased radiodensity compared to the empty control group and higher radiodensities compared to the EHPA group, but this was lower when compared to the Bio-Oss group (Table 1). In axial sections, a complete bridging of the defect was noticed in all the samples from the subjects treated with Bio-Oss, whereas in the sites treated with the composite material, there were only a few radiolucent areas, suggesting good bone regeneration. In the sites treated with EHPA, three to four isolated islands of radiodense areas were noted in the axial sections of the defect, whereas empty control sites appeared radiolucent throughout the defects (Figure 4).

**Table 1:** The bone densities of the treated and untreated sites in all the groups, as shown by ImageJ analysis.

Control group			Eggshell HPA group		Composite group		Bio-Oss group	
Healthy site		Defect area	Healthy site	Defect area	Healthy site	Defect area	Healthy site	Defect area
After 90 days								
1	171.294	69.563	169.356	86.67	165.105	134.57	179.50	138.90
2	171.320	65.263	149.47	95.8	178.25	125.42	143.80	122.28
3	161.020	47.174	161.586	75.949	144.966	141.82	172.54	195.07
4	173.292	50.266	165.08	102.757	159.46	134.573	152.22	145.67
5	173.330	62.24	175.892	107.099	173.55	134.5	178.79	164.20
6	157.593	51.125	172.7	86.672	172.54	104.179	170.845	190.137

#### 3.1.2 Histomorphometric analysis

##### 3.1.2.1 Empty control sites

**Figure 5:**
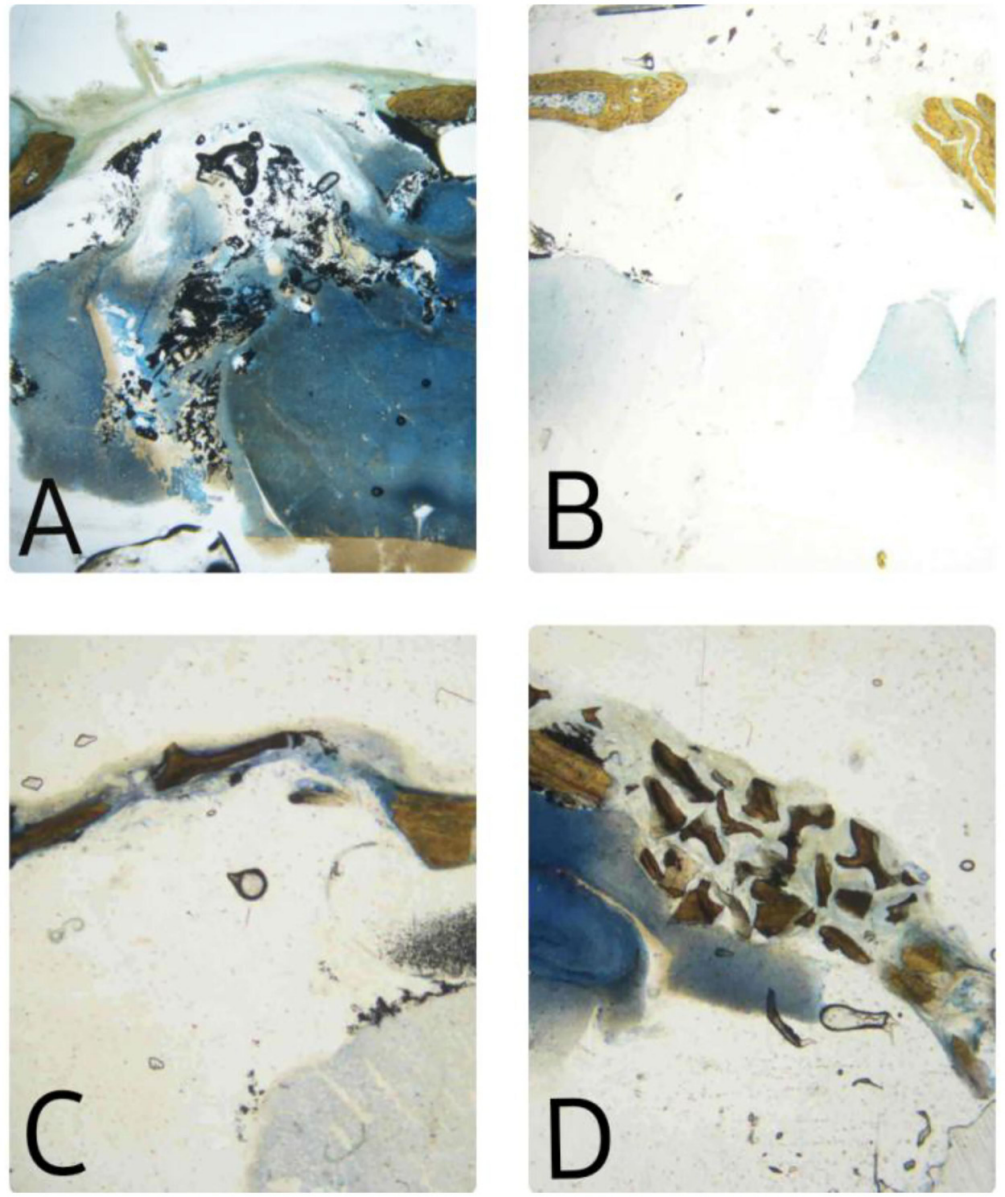
Specimens stained with hot Stevenel's Blue: (**A**) empty control site, (**B**) site treated with EHPA, (**C**) site treated with the composite graft, and (**D**) site treated with Bio-Oss.

**Figure 6:**
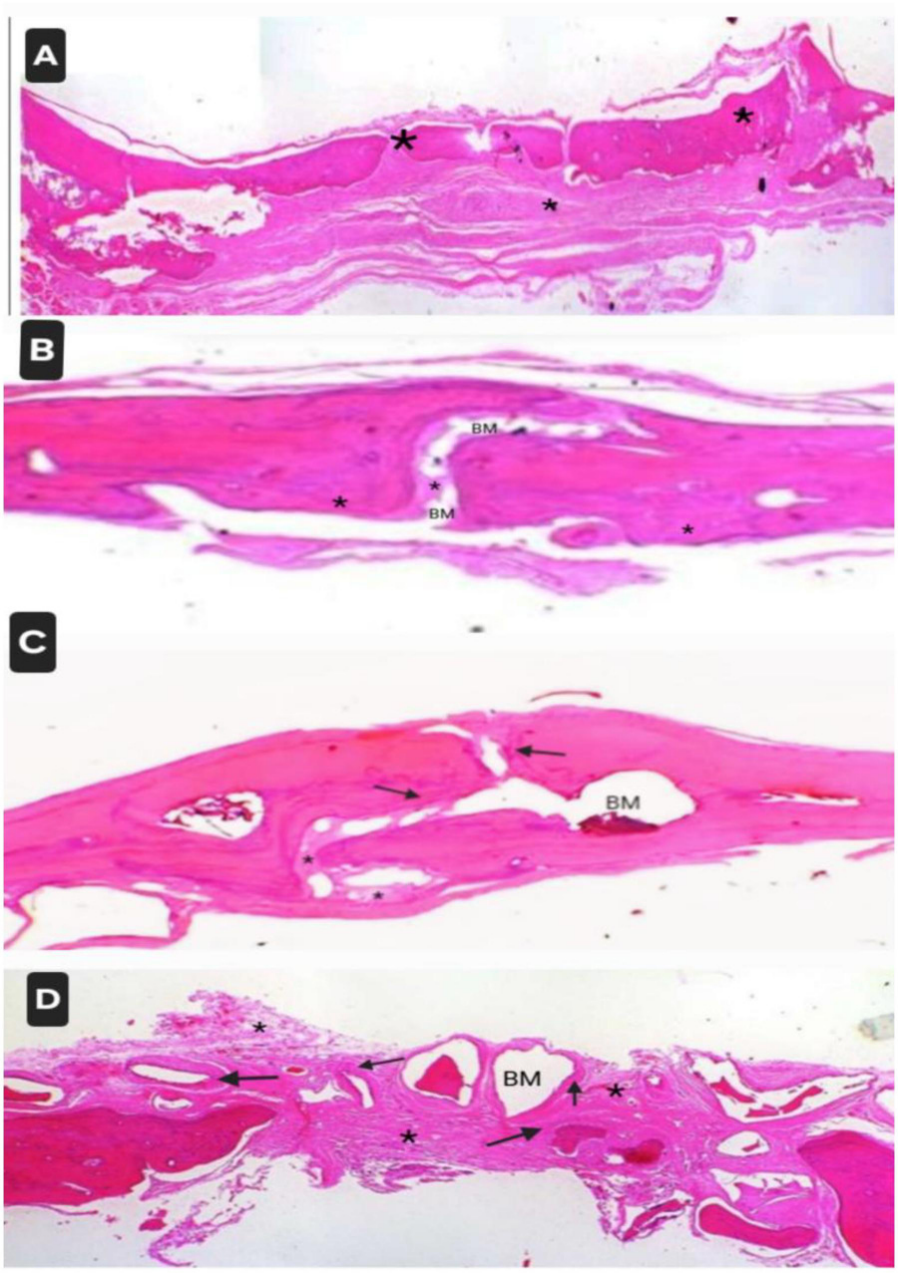
Specimens stained using hematoxylin and eosin: (**A**) empty control site, (**B**) sites treated with EHPA, (**C**) site treated with the composite graft, and (**D**) site treated with Bio-Oss. * denotes fibrous tissue formation. Arrow denotes new bone formation. BM represents residual bone graft material.

Fibrous connective tissues covering the entire calvarial defect areas were noted with calvarial edges on either side. New bone formation was found to be absent in defect sites, and neovascularization was not observed. Moderate mononuclear cell infiltration was found. In a few samples, mild bone formation was noted at the defect margins (Figures 5A, 6A).

##### 3.1.2.2 Sites treated with eggshell-derived hydroxyapatite

Focal areas of connective tissue covering the defect regions with calvarial edges on either side were noted. New bone formation was absent in the middle part but noted near the margins. In most of the samples, isolated residues of the graft material encapsulated within connective tissue were noted. Mild mononuclear cell infiltration was identified (Figures 5B, 6B).

##### 3.1.2.3 Sites treated with composite graft

Focal areas of implant material were observed in the calvarial defect region. Mild connective tissue proliferation was found. In the majority of the samples, new bone formation was noted at the defect margins and within the defect, whereas in a few, it was absent in the middle of the implant site. Neovascularization was observed. Some of the implant fragments encapsulated within the connective tissue were also found. Mild mononuclear infiltration and foreign body giant cells were identified in some of the samples (Figures 5C, 6C).

##### 3.1.2.4 Sites treated with Bio-Oss

Focal areas of multiple bone-fragment-like materials were found in the calvarial defect region. New bone formation was noted at the defect margins as well as within the defects. Mild connective tissue proliferation and encapsulation of fragments were observed at the implant site. Mild mononuclear cell infiltration, macrophages, and foreign body giant cells were observed at the implant site (Figures 5D, 6D).

The osteoconductivity grading of the new bone formation in the defect areas and implanted sites was done semi-quantitatively (19) (Table 2).

**Table 2:** Grading for new bone formation.

Grade	Implant area showing new bone formation
0	No evidence of new bone formation
1	>0 up to 25% of the implant area
2	26%–50% of the implant area
3	51%–75% of the implant area
4	76%–100% of the implant area

A similar grading system was used to evaluate the fibrous tissue formed in the defect sites.

**Figure 7:**
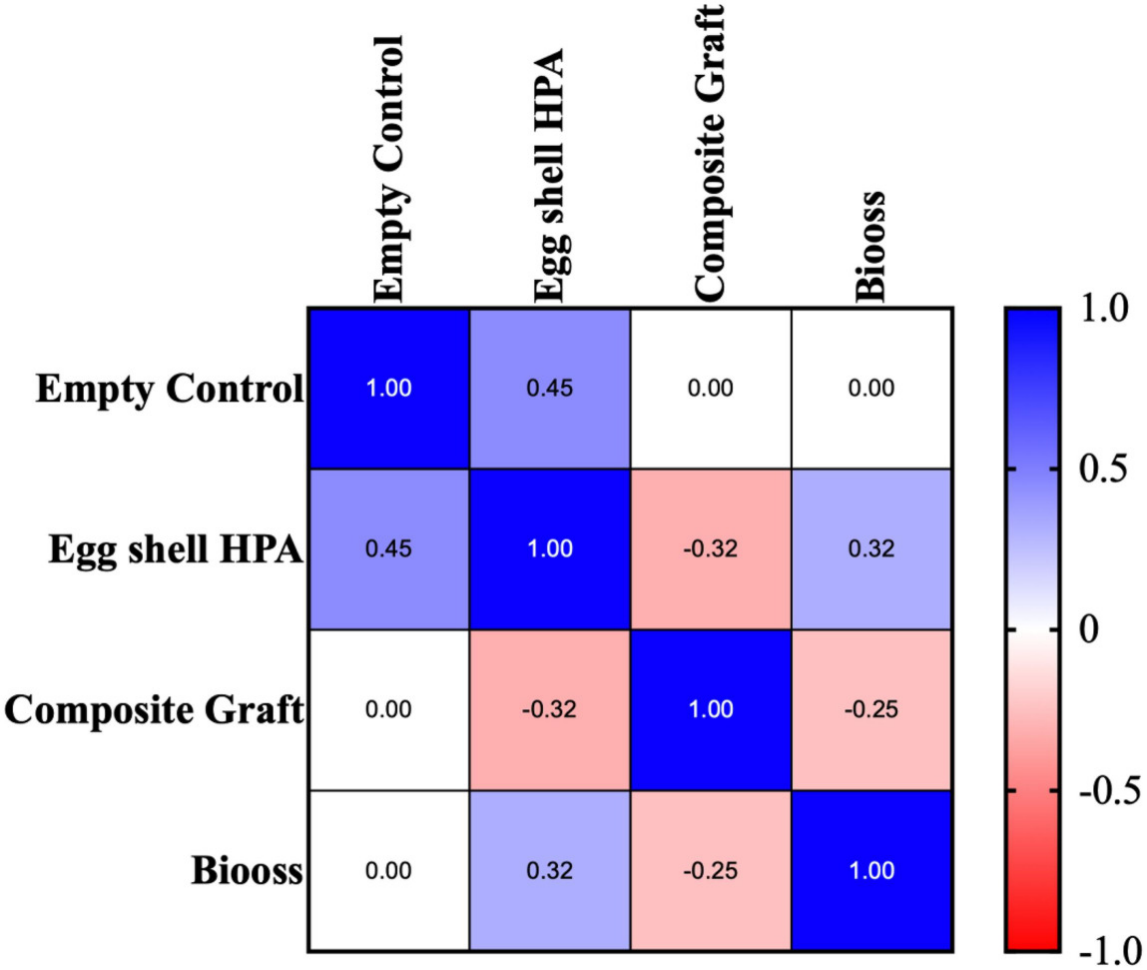
Spearman's correlation of new bone formation in the samples among the groups using the histological data.

Spearman's rank correlation analysis was performed to evaluate the relationship between the treatment groups and histomorphometric outcomes. A significant positive correlation was observed between treatment type and new bone formation, indicating that the groups treated with biomaterials, especially the composite graft and Bio-Oss, consistently demonstrated greater new bone formation (Figure 7).

**Figure 8:**
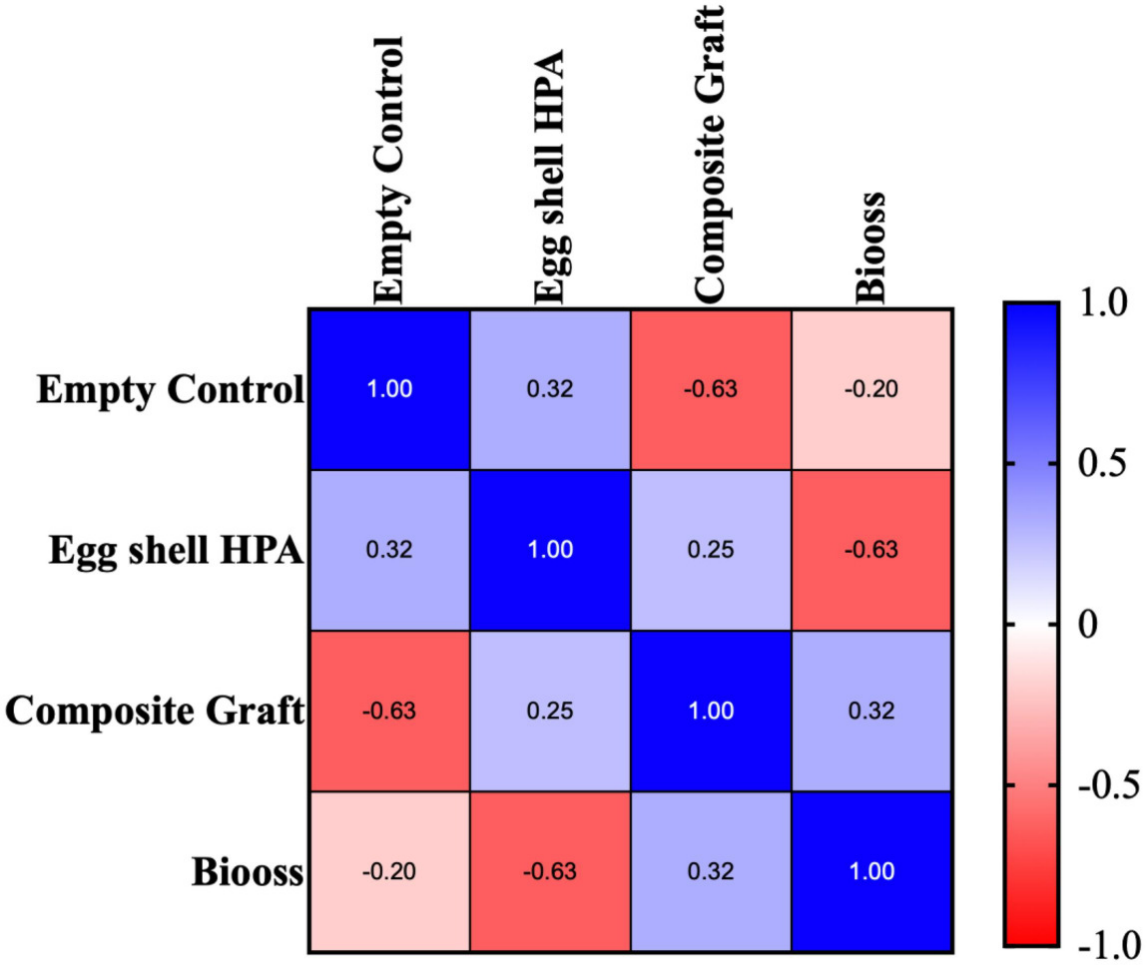
Spearman's correlation of fibrous tissue formation in the samples among the groups using the histological data.

Conversely, fibrous tissue formation showed a significant negative correlation with treatment type, with the empty control group exhibiting the highest fibrous tissue content and the Bio-Oss group the lowest. These correlations substantiate the hypothesis that increasing material osteoconductivity corresponds to improved healing and reduced fibrous encapsulation (Figure 8).

## 4 Discussion

Bone replacement grafts are extensively used to encourage bone formation and periodontal regeneration (20). Hydroxyapatite-based materials have drawn considerable attention in the field of tissue engineering due to their close resemblance to the mineral fraction of natural bone and high cytocompatibility with living tissues. Recently, eggshell-derived hydroxyapatite has emerged as a highly economical bone substitute because of the abundant availability of raw material (21).

Collagen forms molecular scaffolds that provide a framework for tissues and organs. Simultaneously, it has the ability to bind to receptors at the cell surface and modulate their activities (22). Collagen also exhibits excellent biocompatibility, biodegradability, absorbability, and porosity, facilitating directed cell growth and a metabolic interconnection structure, making it one of the most versatile biomaterials (23). The compromised physical properties and rapid degradation rate of collagen restrict its use as an independent biomaterial for osseous regeneration, and justify its need to be combined with other materials with differing properties. Composites based on collagen and hydroxyapatite are the most commonly used bone biomaterials, with the ability to stimulate and substitute skeletal tissue (24).

The regenerative materials that are commercially available have their own shortcomings, as they are either too expensive or do not perform well clinically. The novel composite graft synthesized in this study was derived from two distinct natural raw materials that are usually discarded as waste, easily available, and cost-effective. Hydroxyapatite was synthesized from domestic chicken eggshells and collagen was extracted from fish scales. The composite material was found to have good bone regenerative properties. The incorporation of glycerin improved its consistency and facilitated its use in an injectable form.

### 4.1 Bone formation *in vivo*

#### 4.1.1 Radiographic bone fill

New bone formation in the defect area was examined by using cone beam computed tomography, and the images were analyzed using ImageJ to assess the bone density. The defects that were treated with the composite material or Bio-Oss displayed increased radiodensity and bridging of the defects. The CBCT images confirmed the superior regenerative capacity of the composite graft, which contained eggshell-derived hydroxyapatite and fish collagen, as compared to the empty controls and pure EHPA. The results were statistically significant. However, when compared to the standard commercially available graft, the bone density was found to be marginally lower. Most of the sites treated with EHPA displayed isolated radiopaque areas, which may be due to the residues of unresorbed graft materials or new bone.

Micro-computed tomography (micro-CT) analysis by Kattimani revealed new bone formation at the periphery of the defects in groups filled with synthetic or eggshell-derived hydroxyapatite. In the eggshell nano HPA group, bone growth was statistically significant, and there was a progressive increase in the thickness of bone and density of the matrix, with a decrease in fibrous connective tissue throughout the healing process, compared to the synthetic HPA group (25). Similar observations were noted by Jayasree et al. in another micro-CT analysis, in which they observed increased bone formation in sites treated with eggshell-derived brushite in a rat calvarial defect model (26).

Some of the randomized controlled trials in humans also supported these results. Ganapathy et al. confirmed an increase in bone density values in sites treated with eggshell-derived hydroxyapatite in human periodontal intrabony defects assessed using CBCT (27). Nainoor et al. also found increased radiographic bone density and bone height in the CBCT images of sites treated with eggshell-derived nano hydroxyapatite with platelet-rich fibrin (PRF) compared to decalcified freeze-dried bone allograft with PRF in socket preservation of mandibular molar extraction sockets (28).

However, in our study, the sites treated with the composite graft showed significantly better radiographic bone fill and increased bone density than those treated with pure eggshell-derived HPA and the empty control sites. These radiographic differences were also statistically significant, reinforcing that the composite graft promotes superior regeneration compared to the empty controls and EHPA, although Bio-Oss continues to outperform all materials in terms of radiodensity (*p* < 0.05). When compared to the sites treated with Bio-Oss, the difference in the density of the formed bone was marginal but statistically significant. The results suggest that the novel biomaterial performs fairly well, which is promising for future translational applications.

A combination of EHPA with other materials offered complementary effects and enhanced their regenerative properties. Nelogi et al. found increased bone regeneration and bone density radiographically in sites treated with zinc nanoparticles induced eggshell collagen membrane compared to Healiguide in a rabbit model (29).

The studies by Hatakeyama et al. and Minardi et al. also supported improved bone formation with micro-CT and dynamic CT analyses of sites treated with hydroxyapatite/collagen composites (30, 31).

Another study by He et al. using magnetic resonance imaging (MRI) and micro-CT in rat models also demonstrated enhanced new bone formation with the use of a biomimetic collagen composite matrix-hydroxyapatite scaffold (32). The above results are in agreement with our study, establishing that combining EHPA with other biomimetic materials, especially collagen, results in better bone regeneration.

#### 4.1.2 Histomorphometric analysis

The findings of our study reveal that the empty control sites were mostly covered with fibrous tissue, except in a few samples where new bone formation was noted only near the margins. In the sites treated with EHPA, isolated fragments of bone-like material were noted along with new bone formation restricted to the margins of the defects. In the sites treated with the composite group, new bone formation was noted within the defect and near the edges, with the presence of neovascularization, though a small amount of fibrous tissue was detected. In a few of the samples, new bone formation was absent in the middle of the implant site. The sites treated with Bio-Oss had the greatest thickness of newly formed bone with the least fibrous tissue. The correlation results further support the histological observations. The strong positive Spearman's rho for new bone formation demonstrates that treatment groups using osteoconductive biomaterials were consistently associated with higher bone regeneration. Similarly, the negative correlation for fibrous tissue formation reflects that the more effective graft materials reduced fibrotic healing responses. These statistically significant monotonic associations reinforce the reliability of the group-wise differences identified in the ANOVA and the qualitative histological findings.

Ou et al. found that a weight ratio of 7:3 of E HPA/Coll, resulted in early new bone formation compared to other ratios (3:7, 5:5, and 9:1) (33).

Mashipur et al. observed several foci of spongy and lamellar bone tissue among the callus connective tissue in EHPA-treated sites (34).

Mild mononuclear cell infiltration was noted in all the groups. In the composite group, a few foreign body giant cells were also noted. In the Bio-Oss group, foreign body giant cells and macrophages were observed, as well as the mononuclear cell infiltration at the implant site. Hence, it can be inferred that the composite graft induced a milder inflammatory response compared to Bio-Oss.

Enhanced osteogenic properties and favourable degradation rates were observed by Parisi etal in sites treated with a combination of marine spongin and HPA (35) and Shang et al. in their *in vitro* and *in vivo* analysis with the use of pepsin hydrolyzed tilapia fish collagen/hydroxyapatite composite (36). These findings are in agreement with our study.

Though previous studies have supported the advantages of HPA/COLL composite grafts, this study is unique as it combines eggshell-derived hydroxyapatite and fish collagen, both of which have a natural origin, and have their own environmental and cost benefits, creating a synergy that is more effective than either of the components used alone.

The surgical reentry was only conducted after 90 days. Radiographic bone fill was also not assessed at multiple time points, which can be considered a shortcoming of the study. Further *in vivo* studies and human trials are required to confirm the composite’s osteoinductive properties. The combination of the novel material with other biomimetic materials, such as growth factors, should be studied in the future.

## 5 Summary and conclusion

The present study utilized two novel biomaterials, namely, EHPA synthesized from eggshells and the EHPA/COLL composite graft synthesized from eggshell-derived hydroxyapatite and fish collagen as a bone substitute. The composite graft was prepared by homogenously blending the components and the lyophilization method. The prepared material was mixed with glycerol, which improved the manipulation of the material during its placement in the defect. The novel composite graft was found to have good bone regenerative capacity in rat calvarial defects compared to the empty controls and sites treated with pure EHPA, both radiographically and histomorphometrically. There was only a marginal difference in the radiographic bone density between the composite and Bio-Oss groups. Although the density of the newly formed bone showed slightly lower values compared to the Bio-Oss group, the composite and eggshell-derived graft group showed a lower inflammatory response than the Bio-Oss group. In conclusion, an eggshell-derived hydroxyapatite and fish scale-derived collagen biocomposite can be used as a reliable bone graft substitute in dentistry and orthopedic applications in the future.

## Data Availability

The original contributions presented in the study are included in the article/**Supplementary Material**, further inquiries can be directed to the corresponding author.
