# Supplementary material for: Comparative evaluation of the effectiveness of a novel composite bone substitute synthesized from eggshell-derived hydroxyapatite and fish collagen in bone regeneration of critical-sized calvarial defects in Wistar rats
Source: Front Dent Med. 2026 Jan 12;6:1731880. doi: 10.3389/fdmed.2025.1731880 (PMC12833466; doi:10.3389/fdmed.2025.1731880)
Supplement: Supplementary file 1 [file Datasheet1.pdf]

Fourier Transform Infrared Spectroscopy

FTIR(SHIMADZU) spectra analysis in the range of 4000 to 500 cm^{-1} was employed to characterize the different functional groups present in the material at intensity mode of percentage transmittance . The powdered samples were placed on a sample holder. About 45 scans were done at resolution of 2-4 cm^{-1}

FTIR of EHPA

HA samples exhibited well-defined absorption bands in the infrared spectra [Figure 2a]. The spectra showed the phosphate (PO4)3- group at hydroxyl (OH-), and carbonate (CO3)2- groups at 2363.90. The structural OH- vibrational mode was present in the samples at 3309.46 represented by a broad peak.

FTIR of E HPA

Observed band	Corresponding functional group
3309.46	- OH- stretching
2363	CO – stretching
1646.87	H2O absorbed
1019.83	(PO4)3
869.36	(CO3)2-
555.62	H(PO4)3- bending
598.47	(PO4)3- bending
515.36	(PO4)3- bending

FTIR OF EHPA/Coll Composite

The bands at 3263 cm^{-1} correspond to OH, and N-H groups correspond to 2939 cm^{-1}, 2886 cm^{-1}. CH stretching is observed at 1649 cm^{-1}. 1409 cm^{-1} stretching corresponds to the CO group, and 1034 cm^{-1} represents the phosphate group. There are no intensive bands in the spectra range of 1800-2800 cm^{-1}, a characteristic of all biological materials. The absorption bands at 1409 cm^{-1} and 1649 cm^{-1} correspond to collagen. The peak at 1649 cm^{-1} represents the amide I bend. Two near 6vibrations bands at 2900 cm^{-1} arise from collagen, most probably from the vibration of the OH group.[7]

FTIR of E HPA/ Coll composite

Observed band	Corresponding functional group
3263.75	- OH- stretching
2939.52	N-H
2886.67	N-H
2363	CO – stretch
1649.73	CO(Carboxyl)-stretching, Amide I absorption
1409.77	CO(Carboxyl)-stretching
1034.12	(PO4)3-
554.19	H(PO4)3- bending

Data of the *Invitro* study

FTIR of EHPA | FTIR of Composite

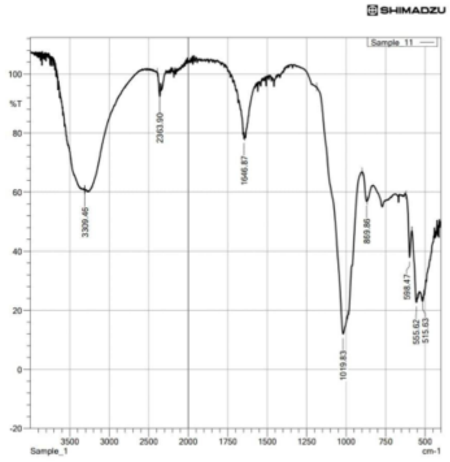

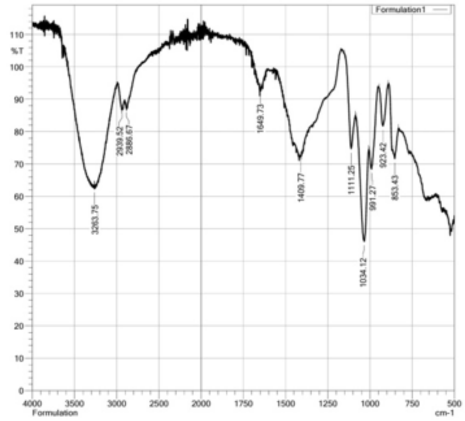

XRD

Rigaku Miniflex 600 (5th gen) with X-ray generation up to 40 k V and current (15 mA) was used here. Nickel filters were incorporated for wide angle measurement (50°- 130°)2 theta value. It facilitates qualitative and quantitative analysis of polycrystalline materials, phase identification and quantification, percent of crystallinity, crystal size and strain, lattice parameter calculation. Types of samples which can be analysed include powder, thin films and metals.

The powdered samples were placed on the sample holder and scanned over 20 range of 10 -80. The diffraction pattern were analysed.

XRD of EHPA

XRD Strongest peak was noticed at 31.54 and 31.68 (925). Other peaks at 33.9 and 39.81 and some minor reflections in the 20 range (600 to 658) were detected. Some minor peaks were also observed at 27.56 (675)

X-ray diffraction spectrum of E HPA-Collagen composite

Strongest peak was recorded at 31.4. The X-ray diffraction pattern of HPA and collagen mixture33 showed strong characteristic patterns of HPA. Pure collagen is amorphous and shows no characteristic peaks. The crystallinity of HPA was not altered significantly by the presence of collagen. The results of XRD patterns include the organic and ionic species. The synthesized compound matched with JCPDS 09-0432 (Joint Committee of Powder Diffraction Standards).

XRD of EHPA

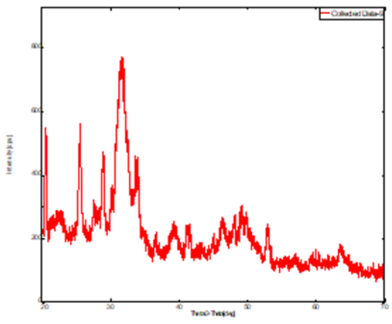

XRD of Composite

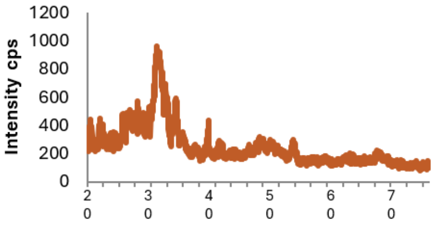

Assessment of cell viability by MTT assay

Cell line and culture condition

L929 murine fibroblast cells were used for the study. They were cultured in Dulbecco's Modified Eagle's Medium (DMEM) supplemented with 10% FBS, 1% glutamine and 1% antibiotic-antimycotic solution. Cells were maintained at 37°C and 5% CO_2 in a humidified atmosphere throughout the experiments.

1. Assessment of cell viability by MTT assay

Cell viability of the test compound was assessed using Methyl Thiazolyl Tetrazolium (MTT) assay (Mosmann, 1983).

Cells were seeded onto 96 well microtiter plate at a seeding density of 5000 cells/well. Allowed it to attach for overnight at 37˚C and 5% CO_2 in a humidified condition. After adherence, different concentration (0, 12.5, 25, 50, 100 & 200 μg/mL) of the sample was added onto the wells and incubated for 24 hrs 37˚C and 5% CO_2 in a humidified condition. After 24 hrs of incubation media was decanted, MTT reagent (1 mg/mL) was added to the wells and incubated at 37˚C for 4 hrs. MTT solution was removed from the wells and formazan crystals formed were solubilised using DMSO and absorbance was recorded at 570 nm using multimode microplate reader (FluoSTAR Omega, BMG Labtech). Percentage of viable cells of the sample d was calculated with respect to untreated cell control.

Reference:

Mosmann, T., 1983. Rapid colorimetric assay for cellular growth and survival: Application to proliferation and cytotoxicity assays. Journal of Immunological Methods 65, 55–63. https://doi.org/10.1016/0022-1759(83)90303-4.99
