# Supplementary figures and images for: Comparative evaluation of the effectiveness of a novel composite bone substitute synthesized from eggshell-derived hydroxyapatite and fish collagen in bone regeneration of critical-sized calvarial defects in Wistar rats
Source: Front Dent Med. 2026 Jan 12;6:1731880. doi: 10.3389/fdmed.2025.1731880 (PMC12833466; doi:10.3389/fdmed.2025.1731880)

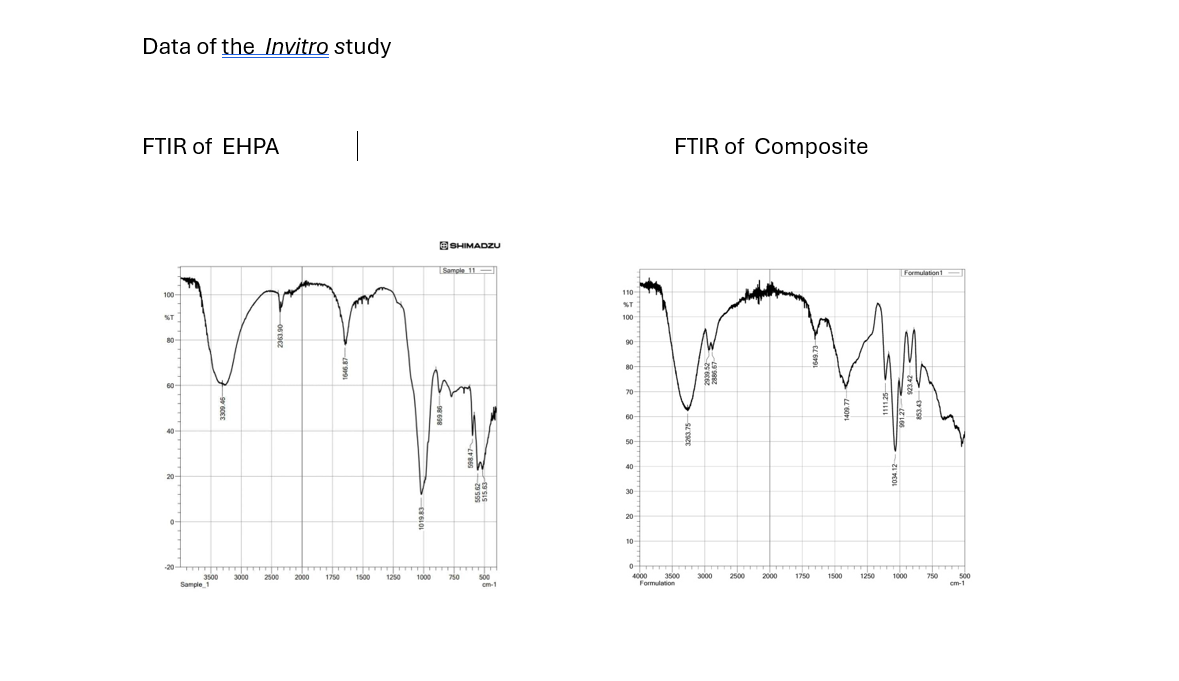

Supplement: Supplementary file 2 [file Image1.png]

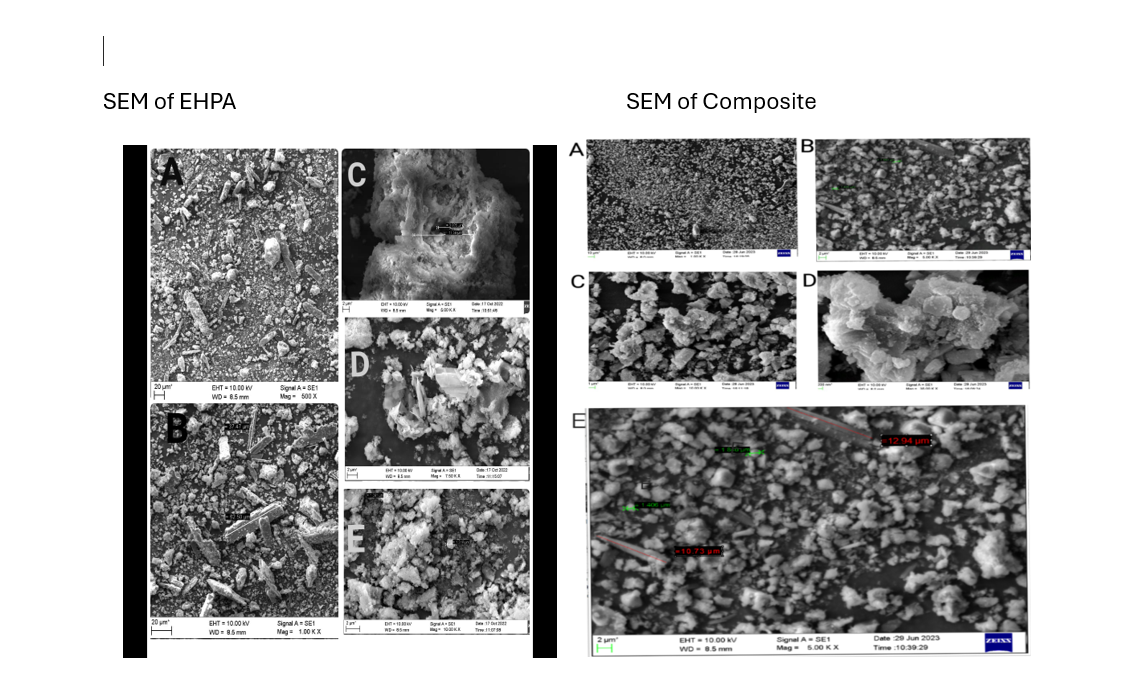

Supplement: Supplementary file 3 [file Image2.png]

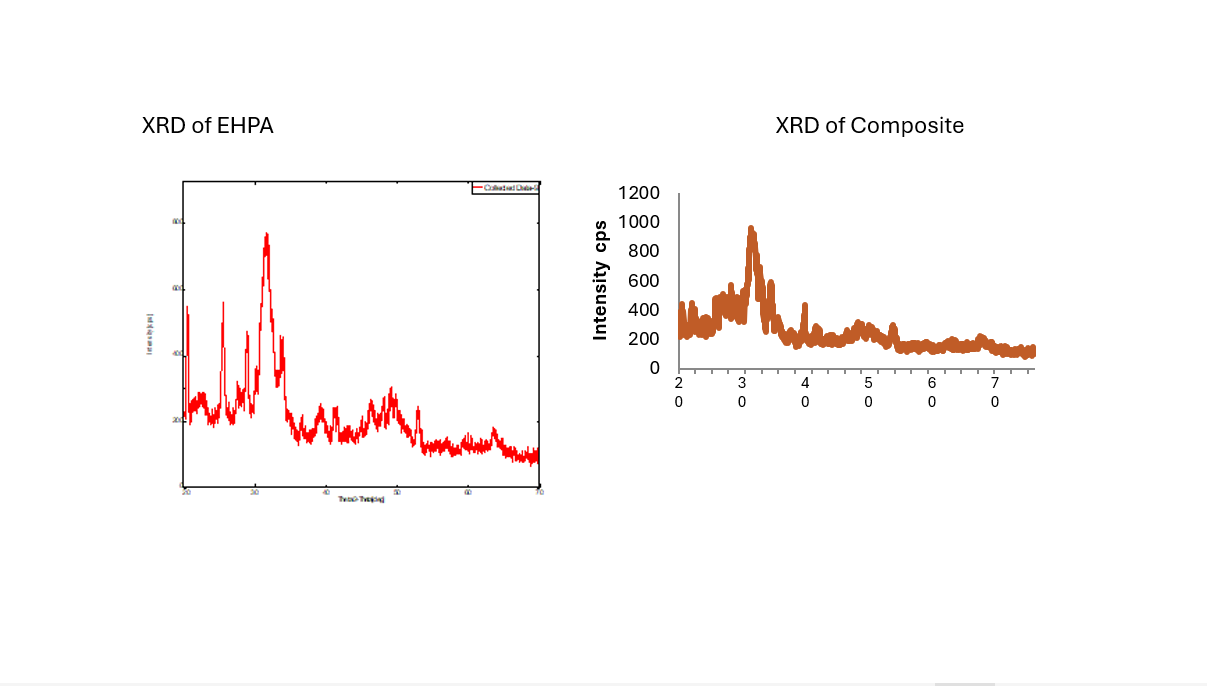

Supplement: Supplementary file 4 [file Image3.png]

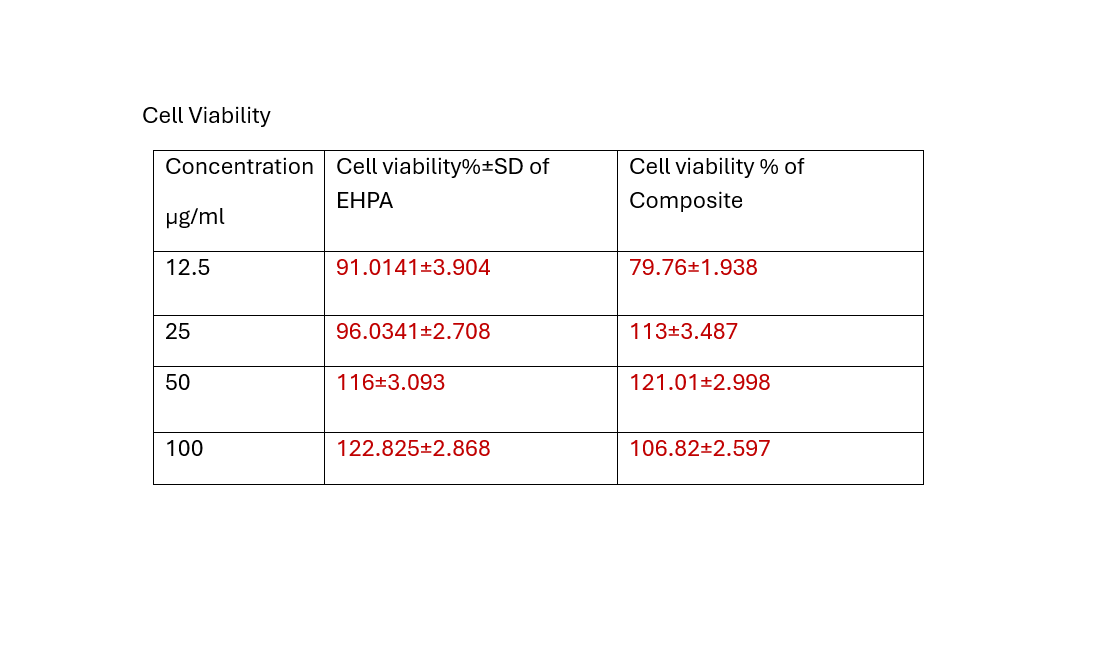

Supplement: Supplementary file 5 [file Image4.png]
